# *microTaboo*: a general and practical solution to the *k*-disjoint problem

**DOI:** 10.1186/s12859-017-1644-6

**Published:** 2017-05-02

**Authors:** Mohammed Al-Jaff, Eric Sandström, Manfred Grabherr

**Affiliations:** 1 Department of Medical Biochemistry and Microbiology, Uppsala University, 75123 Uppsala, Sweden; 2 Bioinformatics Infrastructure for Life Sciences, Uppsala University, 75123 Uppsala, Sweden

**Keywords:** k-disjoint problem, Software, Sequence mining

## Abstract

**Background:**

A common challenge in bioinformatics is to identify short sub-sequences that are unique in a set of genomes or reference sequences, which can efficiently be achieved by *k*-mer (*k* consecutive nucleotides) counting. However, there are several areas that would benefit from a more stringent definition of “unique”, requiring that these sub-sequences of length *W* differ by more than *k* mismatches (i.e. a Hamming distance greater than k) from any other sub-sequence, which we term the *k-disjoint* problem. Examples include finding sequences unique to a pathogen for probe-based infection diagnostics; reducing off-target hits for re-sequencing or genome editing; detecting sequence (e.g. phage or viral) insertions; and multiple substitution mutations. Since both sensitivity and specificity are critical, an exhaustive, yet efficient solution is desirable.

**Results:**

We present microTaboo, a method that allows for efficient and extensive sequence mining of unique (*k*-disjoint) sequences of up to 100 nucleotides in length. On a number of simulated and real data sets ranging from microbe- to mammalian-size genomes, we show that microTaboo is able to efficiently find all sub-sequences of a specified length *W* that do not occur within a threshold of *k* mismatches in any other sub-sequence. We exemplify that microTaboo has many practical applications, including point substitution detection, sequence insertion detection, padlock probe target search, and candidate CRISPR target mining.

**Conclusions:**

microTaboo implements a solution to the *k-*disjoint problem in an alignment- and assembly free manner. microTaboo is available for Windows, Mac OS X, and Linux, running Java 7 and higher, under the GNU GPLv3 license, at: https://MohammedAlJaff.github.io/microTaboo

**Electronic supplementary material:**

The online version of this article (doi:10.1186/s12859-017-1644-6) contains supplementary material, which is available to authorized users.

## Background

There are several areas in bioinformatics and biomedical research that benefit from identifying short sub-sequences among a (large) pool of reference sequences that are as unique as possible, i.e. the next most similar sub-sequence differs by a given number of mismatches or more. For example, in infection diagnostics, pathogen strains can be identified via padlock probes [1], which are highly target sensitive oligonucleotides (30–100 nt in length). However, the specificity of this approach depends on the probe design: since the process is prone to tolerating single mismatches, specificity is increased when using sequences that differ by more than a single mismatch from any other site. Another example constitutes sequence incorporation detection [2]: genomes undergo change through various means, among these through sequences insertions (e.g. phage/viral incorporation), deletion, and point mutations. The phenotypic effects of these range between deleterious, neutral and beneficial to an individual’s fitness as in the case of mutations resulting in antibiotic resistance. Genome alterations by direct sequence incorporation also play a functional role; a prime example is the Clustered regularly interspaced short palindromic repeats (CRISPR) antiviral defense system of many prokaryotes. There already exists software that is able to find putative regions which could be a result of sequence insertions, e.g. CRISPR-finder [3] and RetroTector [4], however, these rely heavily on a priori knowledge, often niche to the sequence type desired to be detected. Alternatively, a generic method based on exact-matches would provide optimal sensitivity, but lacks specificity and will potentially report a number of false positives, in particular if the input sequences are raw sequence reads that contain errors, and/or if the strain or organism is different from the reference genome. A third example in which target design benefits from a stringent definition of uniqueness is the identification of CRISPR target sites for genome editing [5]. It has been shown that unintended off-target editing can be reduced by designing CRISPR guide-RNA to comprise sequences that differ by more mismatches from any site in the rest of the genome [6].

Mathematically, the problem of identifying a set of sequences or strings of length *W* that differ by at least *k* mismatches compared to any other sequence in a set, termed *k-disjoint*, is defined as follows: given two sets *A* and *B* containing sequences of length *W*, find all sequences *X* in *A* such that *X* is more than *k* mismatches away from any sequence *Y* in *B*. This formulation of the problem essentially finds the so-called *k*-disjoint set of *A* and *B*. An alternative formulation is: given two integer values *W* and *k* as well as two strings *P* and *T* with lengths *n* and *m*, respectively, the *k*-disjoint problem consists of finding all positions *i* (1 ≤ *i* ≤ *n*) which have the property that a *W* length substring of *P* beginning at *i* is at a Hamming distance greater than *k* away from any *W* long substring of *T*. The complement of the *k*-disjoint is the *k*-intersection. The *k-*disjoint problem is closely related to the *k*-difference primer problem [7].

Conceptually, *k*-disjoint is an extension of the exact-match problem, since for *k* = 0, the *k*-disjoint problem collapses into identifying truly unique sequences, even if they only differ by one single letter. For this much simpler problem, common solutions include the analysis of *k*-mers, short sub-sequences of a fixed size *k*. There, simple lookup, hash-tables, or suffix arrays provide efficient solutions, with a number of implementations available, among them bloom filter based methods and suffix array based programs, such as Jellyfish [8], and Tallymer [9]. The resulting counts and *k*-mer spectra can thus be used to estimate repeat content or heterozygosity in a genome or sequence reads [10, 11]. In addition, exact-match methods can also serve as seed finders to guide sequence alignment programs (for a review, see [12]), which are then able to extend sequence comparisons into regions in which mismatches occur. In principle, this approach allows for computing “inverse alignments”, i.e. to first perform alignments, and then exclude regions that show sequence similarity above a given threshold. However, since many sequence aligners typically include a dynamic repeat masking step when finding seeds for computational efficiency, these methods are not exhaustive in the sense of looking for evidence of absence (no match at a given distance or higher), rather than evidence of presence (match at a given distance or lower), and do thus not guarantee that no false positives are found.

Here, we present microTaboo, a general, exhaustive, and efficient solution to the *k*-disjoint problem based on a mismatch matrix and a dictionary tree for fast lookup and search (see Implementation). The output of this method, all sequences that avoid “taboo”, can either be used directly, or serve to pre-filter data for software for specific tasks, such as CRISPR-finder or SMS [13]. microTaboo is a generalization of the work done by Mattisson et al. 2016 [14]. Briefly, the work done in Mattison et al. was a rough and *ad hoc* solution to a niche problem concerning the identification of unique sequences common amongst coagulase negative *Staphylococci*. Here, we have generalized, refined, and optimized the algorithm and data workflow, as well as packaged the solution into a fully functional software package, microTaboo.

## Implementation

### Algorithm, architecture and workflow

microTaboo requires the following inputs: i) two directories representing queries *A* and targets *B*, each containing one or more FASTA files; ii) an integer value, *W*, indicating the sequence lengths to be found in *A;* iii) an integer value *k* representing the mismatch threshold; and iv) a parameter (‘d’, ‘i’ or ‘a’) specifying whether the results should present only the *k-*disjoint, the *k-*intersection, or both. microTaboo outputs a two column file for each FASTA file in the query directory, containing sequences satisfying the input parameters, together with their sequence-relative positions.

**Fig. 1:**
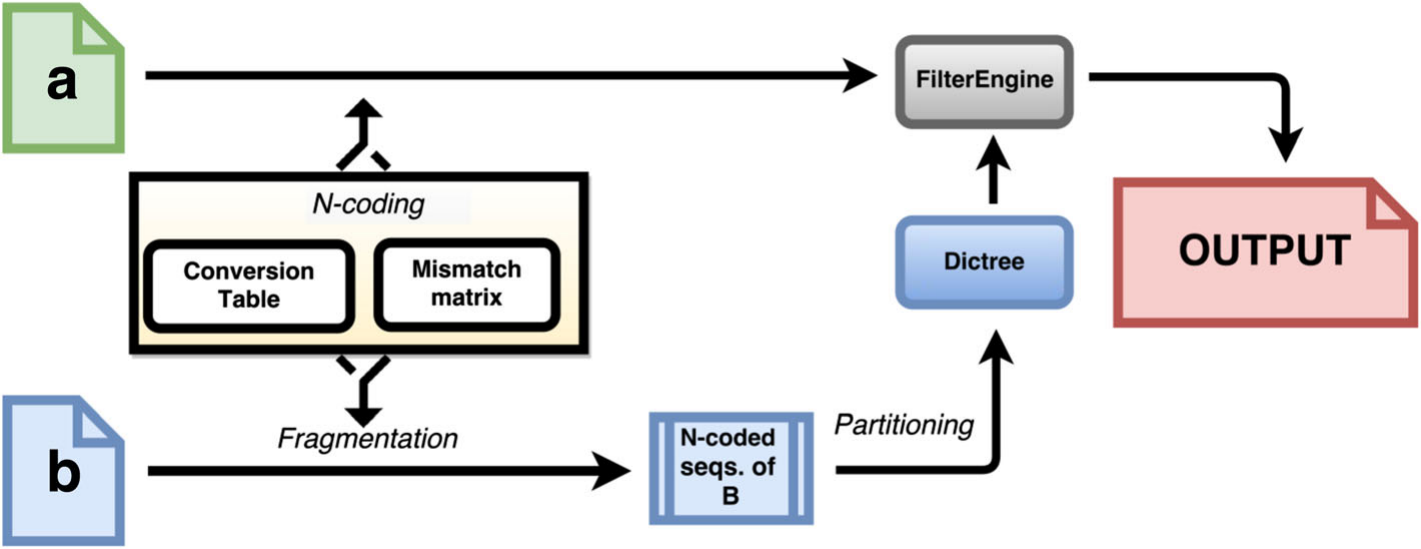
Data and process flowchart of microTaboo. The flowchart shows the main modules, data and processes involved in the internal algorithm of microTaboo. *A* and *B* (green and blue documents) represent either a single or multiple FASTA-files. For a user-specified given value of the sequence length *W,* all *W-long* substrings in *B* are converted into their respective N-code vectors, out of which a list of all such vectors in B is outputted. This list then is used to construct a *Dictree* (a hybrid dictionary-tree-list data structure) which is fed into the *filter engine module* responsible for filtering the all *W-*long substrings in A (after N-code conversion) and providing an output file contacting either the *k*-disjoint set, *k-*intersection or both

**Fig. 2:**
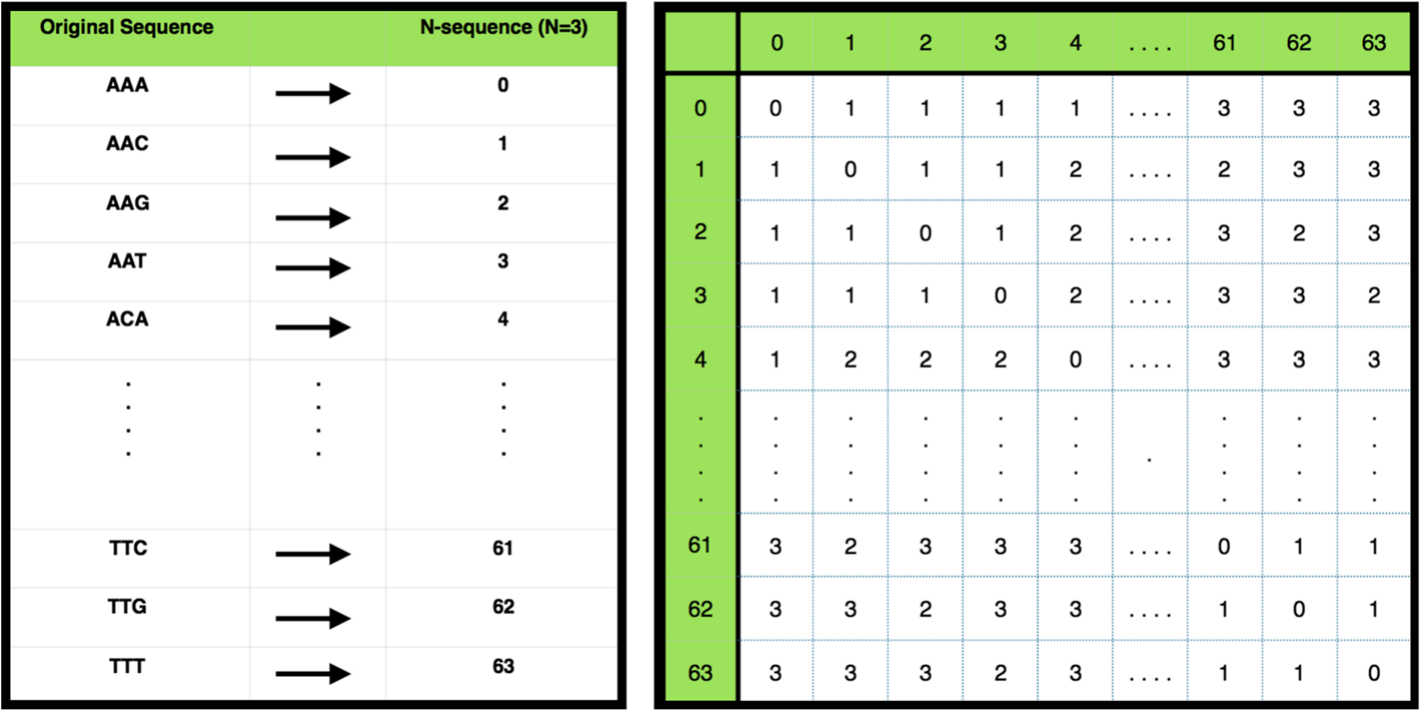
N-code conversion table and Mismatch matrix. The N-code conversion table & the mismatch matrix for *N* = 3. (left sub-figure) Concept of N-coding with *N* = 3 and only using nucleotides (A, C, G, T). Every unique sequence is assigned a unique number in lexicographical ascending order. (Right) Visualization of a mismatch matrix in N-code format where *N* = 3 and only using nucleotides (A, C, G, T). Each cell in the matrix contains the Hamming distances between the respective row and column element, i.e. the sequence or N-code value represented there. For example, the sequence “AAA” → <0 > and the sequence “AAG” → <2 > are at a Hamming distance of 1 away from each other as in the cells (0,2) and (2,0). Meanwhile, the distance between sequence “AAA” and “TTT” is 3 → <63 > as in cell (0, 63) and (63,0)
ii) *Fragmentation* extracts all sequences of length *W* (*W* is a multiple of *N*) in *B,* reverse complement strand included*,* and converts them into a list of vectors of length *W/N* using N-coding scheme*.* The output of this process is a list of N-coded sequences.
iii) *Partitioning* divides the above list into *sub-lists* with respect to the first two elements of each vector, reducing comparisons. The output of this process is a hybrid dictionary-tree-list data structure, called here a *Dictree,* which is then fed into the *filter engine* module (see below). The Dictree can be viewed as being an indexed list, where all *W-*long sequences (in N-code format) from the B FASTA-files are stored and indexed with respect to their first two vector elements. For instance, all N-code vectors of the form *<62, 3,… >* would be placed into the sub-list with the address: *<62, 3 >* .
iv) *Filter engine search* is the module that filters sequences in *A* against the Dictree of *B* through their respective *W/N* vectors. That is, the filter engine sequentially extracts all *W* long substrings in A, N-codes these and searches the Dictree of B for a match. Thus, querying a vector of the same form for *k = 0* in the *Dictree* requires merely a comparison to the list with exact indices as an exact match to the query vector could theoretically only exist in that particular list address. For *k > 0*, microTaboo looks into multiple lists until either the number of mismatches exceeds *k*, or all sub-lists that could contain a match has been searched in and not found. Using the mismatch matrix, the addresses of all sub-lists that could possibly contain a sequence within *k*-mismatches of a given sequence in N-code format, *X = {x*_*1*_ *, x*_*2*_ *, x*_*3*_ *, x*_*4*_ *, …}*, is obtained in the following way:

The underlying algorithm that powers mircoTaboo involves several steps (see Fig. 1):

i) *N-coding* first maps all sequences of size *N* onto unique numbers, and then pre-computes a mismatch matrix by calculating the number of mismatches between two N-sequences, allowing for fast look-up; N-coding converts all unique sequences of a fixed length, N, to a unique number (see Fig. 2). This enables us to represent a sequence of length *W* as a vector consisting of *W/N* elements, where *N* can take the value 3, 4, or 5, requiring *W* to be a multiple thereof. Given that microTaboo works with a five letter DNA alphabet which includes a canonical unknown base, to avoid excessive memory usage, which grows with *O*(5^2N^), *N* is limited to 5. If *W* has multiple delimiters, microTaboo chooses the largest value for *N*. Next, a mismatch matrix *M* is constructed, the elements *M(i,j)* being the number of mismatches between two N-code sequences. Both N-coding sequences and the mismatch matrix enable us to calculate mismatches between two sequences more efficiently, such that the mismatches between two *W*-long sequences can be calculated in *W/N* look-ups in the mismatch matrix.

Address 1 = *i*, such that: *M*(*x*_1_, *i*) ≤ *k*

Address 2 = *j*, such that: *M*(*x*_2_, *j*) ≤ *k* − *M*(*x*_1_, *i*)

Thus, only the lists in the Dictree with addresses *i* and *j* that satisfies these conditions could possibly contain a sequence within *k* mismatch from the query sequence *X*.

### Computational complexity of microTaboo

There are two computationally intensive procedures involved when running microTaboo, namely, constructing the Dictree, and searching the query organism in the Dictree. To evaluate their respective complexities, we define the following: word length (*W*), N-code size (*N*), genome length of query organism (*L*_*A*_) and genome length of “taboo” organism (*L*_*B*_). Construction of the Dictree is done in two steps: extracting and converting all words of length *W* from the “taboo” organism to *N*-code format and inserting the *N*-code vectors into the Dictree. The number of operations required to convert a single word into an *N-*code vector is:

1 $$ \frac{W}{N}{C}_1 $$

Where C_1_ is some constant. Inserting the resulting *N-*code vector in the Dictree takes a constant number of operations, denoted C_2_. Therefore, the amount of operations needed for populating the entire Dictree with all encoded sequences of the “taboo” organism is:

2 $$ \left[\frac{W}{N}{C}_1 + {C}_2\right]{L}_B $$

The second computationally heavy procedure is searching the Dictree for all words of length *W* in the query organism so as to filter out those that do not meet the criteria for the *k-disjoint* problem with specified parameters. Assuming that sequences of *N*-long bases are equally frequent in the genome of the “taboo” organism will imply that each sub-list of the Dictree will contain roughly *L*_*b*_/5^2*N*^ elements (note that this makes the storage complexity of microTaboo $O\left({L}_b\frac{W}{N}\right)$). The denominator, (1/5^2^) comes from the fact that microTaboo uses a five letter DNA alphabet and that we have (5^*N*^)^2^ leafs in the Dictree, each pointing to a sub-list with address *i, j.*

Since any *W* long sequence is first converted into a vector in N-code format, which takes $\frac{W}{N}{C}_1$ operations. This vector is then processed by the filter engine and pointed to the corresponding sublist which takes C_2_ operations. Given that this sub-list contains *L*_*b*_/5^2*N*^ elements, in the worst case, an equal number of vector-to-vector comparisons will be made. Each vector-to-vector comparison will consist of $\frac{W}{N}$ look-ups in the mismatch matrix. Given that a look-up takes a constant number of operations C_3_, the complete computational complexity for one word is

3 $$ \frac{L_B W}{5^{2 N} N}{C}_3 + \frac{W}{N}{C}_1 $$

The computational operation burden to filter a query organism against a “taboo” organism is:

4 $$ {L}_A\left[\frac{L_B W}{5^{2 N} N}{C}_3 + \frac{W}{N}{C}_1\right] $$

Combining the complexities for the search and the construction of the Dictree gives:

5 $$ \left({L}_A+{L}_B\right)\frac{W}{N}{C}_1 + {L}_B{C}_2+{C}_3\frac{L_A{L}_B W}{N{5}^{2 N}} $$

In practice, L_A_ and L_B_ are the dominating factors in terms of size. Therefore, the term involving the product of these would be the determining factor, making the theoretical complexity:

6 $$ O\left(\frac{L_A{L}_B W}{N{5}^{2 N}}\right) $$

However in practice, the denominator *N*5^2*N*^ gives a reduction in the number of computations needed resulting in vastly reduced runtimes, e.g. for any word that is divisible by 5, *N* becomes 5 which makes the denominator be 5^11^. Taking into account *k* mismatches, we have empirically determined that microTaboo scales in the following fashion: *O*(10^*k*^).

## Results and discussion

### Infection diagnostics with padlock probes

We identified padlock probe targets unique to *Escherichia coli* O157 Sakai [15], under the condition that the targets are not present within *k* = 5 mismatches of any *W* = 30 bp long sequence in 25 other bacteria, including four other *E. coli* strains (the “taboo” organisms, Additional file 1: Table S1). We identified 46,461 candidate regions not present in the 25 other strains, which could thus serve as potential targets to uniquely identify this strain (see Additional file 2: Section 1.1.).

### Point-mutation detection

We simulated a run which aimed at detecting substitution mutations between closely related strains, such as in gaining drug resistance, by randomly generating substitutions in three genomes of different sizes (see Additional file 2: Section 1.2 and Additional file 3: Table S2): the *Tobacco leaf curl Japan virus* [16] (*TbLCJV,* ~2.5 kbps), *E. coli* (~5.5Mbps), *Saccharomyces cerevisiae* [17] (12Mbps), and *Candida albicans* [18] (~14.3Mbps). We randomly selected 5 positions per chromosome in each organism and altered the bases accordingly: A<>G and C<>T. With *k* = 0 and *W* = 30, microTaboo successfully recovered all mutated sites (see Additional file 2: Section 1.2.)

### Detection of sequence inversions

We simulated a scenario in which local inversions are to be detected, based on the same organisms as above (*TbLCJV*, *E. Coli* and *S. cerevisiae*, see Additional file 4: Table S3). For each organism, we altered their genomes by inverting three 80 bp long sequences at positions 26,480 (*E.coli*), 1120 (*TbLCJV*), and three 60 bp long regions on chromosomes 1, 2, and 5 in *S. cerevisiae*. microTaboo detected all the sequences in and around the vicinity of the inverted regions. We repeated the experiment by introducing additional point substitutions and achieved identical results (see Additional file 2: Section 1.3.1).

### Detection of viral incorporation

Here, we inserted four regions of size 70 bps of *TbLCJV* into the *S. cerevisiae* genomes into chromosomes 1, 2, 3 and 4. In addition to inserting the regions, we introduced a number of base alterations in the following way: one region was left intact whilst in the others, we introduced base alterations in 1, 2 or 3 positions respectively. Running microTaboo with parameters *W* = 70 and *k* = 3, mircoTaboo reported the insertion sites and their vicinities (see Additional file 2: Section 1.3.2).

### Candidate CRISPR-target mining

We applied microTaboo to find unique CRISPR target sequences of length *W* = 20 bp in the genomes of *C. albicans* strain S288C, *D. Melanogaster* [19]*,* and chromosome 16 of *M. Musculus* [20] against the entire mouse genome*.* Varying *k* = 0, 1 and 2, the results are listed in Table 1, reporting both the genomic terretory covered by the sites, as well as the fraction of unique sequences (in parentheses). While the fraction of target sites only drops slightly with increasing *k* to 2 in the small genome of *C. albicans*, this drop is more pronounced in *D. melanogaster*, and falls from more than 80% to less than 5% in mouse, leaving less than one percent of the mouse genome for targeting at this stringency. We repeated this analysis at *k* = 2 on mouse chromosomes 9 and 18 with similar results, 0.6% and 0.5% respectively (see also Additional file 2: Section 1.4).

**Table 1:** Fraction of unique sequences

Organism	% k = 0	% k = 1	% k = 2
*C. albicans*	96.0 (97.4)	91.2 (96.6)	63.7 (94.2)
*D. melanogaster*	92.3 (94.0)	83.9 (92.9)	40.7 (85.9)
*M. musculus*	73.3 (83.0)	32.9 (72.7)	0.5 (4.3)

Listed are the fraction of 20 (*W* = 20) nucleotides long sequences and the genomic territory covered (in parentheses) for *k* = 0, 1, 2 on *C. albicans*, *D. melanogaster* and *M. musculus*. For each run, copies of the files containing the genome for the organism of interest were placed both in the query folder and the “taboo” folder. For the mouse genome, only the genome file for chromosome 16 was placed in the query folder

### Comparison with an inverse alignment approach and a suffix array

To assess the relative run-time and coverage efficiency of microTaboo, we compared the runtime performance and sensitivity of microTaboo against an inverse BLAST+ 2.4.0 search on two random sequences of length 10 kb and 4 Mb, and a ~10 kbps section of the *Enterobacteria phage lambda* [21] phage against the ~4.6Mbps *Escherichia coli K12* [22] genome. On one core of a 64-bit Intel-Core i7-4720HQ machine, microTaboo was up to 61x faster than BLAST, depending on *k* and *W*, in addition to finding more sequences. The fact that microTaboo had a larger coverage is likely attributable to being an exhaustive algorithm (see Additional file 2: Section 2.1 and Additional file 5: Table S4). We next compared the performance to a suffix array, as implemented in the sequence alignment software Cola [23], adjusting the -S parameter, which determines the minimum length of exact sub-matches before giving up the search, for different combinations of *W* and *k* as *S* = *W*/(*k* + 1), following the pigeon hole principle. While the suffix array runs efficiently with larger values of *S* (20 and up), albeit slower than microTaboo in all but one case (*W* = 100, *k* = 5), performance dramatically drops with lower values (Additional file 6: Table S5), since the number of regions to be considered for an exhaustive search increases exponentially. We note that for *S* < 10, which corresponds to e.g. *W* = 40, *k* = 3, we terminated the program after two CPU hours.

### Comparison with exact string-matching algorithms

Since microTaboo outperforms a suffix array even for *k* = 0, we sought to compare the method to exact string matching methods, namely: Rabin-Karp [24], Knuth-Morris-Pratt [25] and Boyer-Moore [26]. On the *Enterobacteria phage lambda* phage *Escherichia coli K12* data set described above, we found that Boyer-Moore was more efficient than the suffix array, Knuth-Morris-Pratt, and Rabin-Karp methods, but at least two to three times slower than microTaboo (see Additional files 2: Section 2.2, and Additional file 7: Table S6).

### Multi-threading and performance

Since the bulk of the computations in microTaboo are multithreaded, we tested the performance gain of running many cores. The reduction in wall clock time is sub-linear with the number of cores (Table 2.) The organisms used for this run were *Enterobacteria phage lambda* as query organism and *Escherichia coli K12* as “taboo” organism.

**Table 2:** Runtime scaling over multiple cores

#cores	Time (s)	Speed up
1	4271	N/A
2	2305	1.85
3	1726	2.47
4	1576	2.71
6	1098	3.90
8	1066	4.00
10	850	5.02

Runtime of microTaboo for different number of cores where all other parameters were fixed. Speed up factor is calculated compared to runtime for a single core. *Enterobacteria phage lambda* was used as query organism and *Escherichia coli K12* was used as “taboo” organism. There parameters used were *W* = 60 and *k* = 3 for all runs

## Conclusions

We present microTaboo, a fast, efficient, and general tool for directly solving the *k*-disjoint problem for short (<100 nt) sequences and *k* ranging from 0 to 5. In a microbial genome, we show that the method is capable of quickly finding padlock probes that allow for uniquely identifying a specific strain, and in three simulated experiments, we demonstrate that microTaboo can identify mutations, inversions, and insertions, even in presence of single-point mutations. For pre-filtering regions that could serve as CRISPR targets at different stringencies of uniqueness, we applied microTaboo to a fungal, fly, and mammalian genome. While the genomic territory at three mismatches leaves only less than 1% of the genome accessible to genome editing at high stringency, examining this fraction as a function might be useful to predict the number of expected off-target hits.

The software is available for the Windows, MAC OS and Linux operating systems and runs both on standard laptop computers for smaller tasks, as well as larger computer clusters for more profound tasks. We also demonstrate that microTaboo can be used for several applications and that it serves especially well as a powerful pre-filtering tool for further analysis of sequence data.

## Additional files

Additional file 1: Table S1. Padlock Probe target search. (DOCX 119 kb)

Additional file 2: Section 1 - Results, Section 2 - Runtime Comparisons. (DOCX 27 kb)

Additional file 3: Table S2. Point mutation detection. (DOCX 39 kb)

Additional file 4: Table S3. Inversion detection and virus incorporation. (DOCX 54 kb)

Additional file 5:Table S4. Runtime comparisons -microTaboo vs. BLAST and a suffix array method. (DOCX 39 kb)

Additional file 6: Table S5. Result coverage comparison — BLAST vs microTaboo. (DOCX 86 kb)

Additional file 7: Table S6. Runtime comparisons for exact string matching algorithms. (DOCX 50 kb)

## Abbreviations

CRISPR: Clustered regularly interspaced short palindromic repeats
