# Supplementary material for: microTaboo: a general and practical solution to the k-disjoint problem
Source: BMC Bioinformatics. 2017 May 2;18:228. doi: 10.1186/s12859-017-1644-6 (PMC5414201; doi:10.1186/s12859-017-1644-6)
Supplement: Supplementary file 1 — Padlock Probe target search. (DOCX 119 kb) [file 12859_2017_1644_MOESM1_ESM.docx]

Additional file 1: Table S1. Padlock Probe target search

| **Query organism** | **Accession**  **number** | **Web link** |
| --- | --- | --- |
| Escherichia coli O157:H7 str. Sakai Chromosome | NC_002695.1 | https://www.ncbi.nlm.nih.gov/nuccore/NC_002695.1 |
| **Target Organisms** |  |  |
| Escherichia coli Xuzhou21 [2] | NC_017906.1 | https://www.ncbi.nlm.nih.gov/nuccore/NC_017906.1 |
| Escherichia coli 042 [3] | NC_017626.1 | https://www.ncbi.nlm.nih.gov/nuccore/NC_017626.1 |
| Escherichia coli O83:H1 str. NRG 857C [4] | NC_017634.1 | https://www.ncbi.nlm.nih.gov/nuccore/NC_017634.1 |
| Escherichia coli O104:H4 str. 2011C-3493 [5] | NC_018658.1 | https://www.ncbi.nlm.nih.gov/nuccore/NC_018658.1 |
| Escherichia coli O111:H- str. 11128 [6] | NC_013364.1 | https://www.ncbi.nlm.nih.gov/nuccore/NC_013364.1 |
| Escherichia coli 0127:H6 E2348/69 [7] | NC_011601.1 | https://www.ncbi.nlm.nih.gov/nuccore/NC_011601.1 |
| Escherichia coli O145:H28 str. RM12761 [8] | NZ_CP007133.1 | https://www.ncbi.nlm.nih.gov/nuccore/NZ_CP007133.1 |
| Escherichia coli O157:H7 str. EC4115 [9] | NC_011353.1 | https://www.ncbi.nlm.nih.gov/nuccore/NC_011353.1 |
| Pseudomonas aeruginosa PAO1[10] | NC_002516.2 | https://www.ncbi.nlm.nih.gov/nuccore/NC_002516.2 |
| Streptococcus pyogenes M1 GAS [11] | NC_002737.2 | https://www.ncbi.nlm.nih.gov/nuccore/NC_002737.2 |
| Streptococcus pneumoniae R6 [12] | NC_003098.1 | https://www.ncbi.nlm.nih.gov/nuccore/NC_003098.1 |
| Enterococcus faecalis V583 [13] | NC_004668.1 | https://www.ncbi.nlm.nih.gov/nuccore/NC_004668.1 |
| Listeria monocytogenes str. 4b F2365 [14] | NC_002973.6 | https://www.ncbi.nlm.nih.gov/nuccore/NC_002973.6 |
| Klebsiella pneumoniae subsp. pneumoniae NTUH-K2044 [15] | NC_012731.1 | https://www.ncbi.nlm.nih.gov/nuccore/NC_012731.1 |
| Citrobacter koseri ATCC BAA-895 [16] | NC_009792.1 | https://www.ncbi.nlm.nih.gov/nuccore/NC_009792.1 |
| Acinetobacter baumannii ACICU [17] | NC_010611.1 | https://www.ncbi.nlm.nih.gov/nuccore/NC_010611.1 |
| Micrococcus luteus NCTC 2665 [18] | NC_012803.1 | https://www.ncbi.nlm.nih.gov/nuccore/NC_012803.1 |
| Enterobacter cloacae subsp. cloacae ATCC 13047 [19] | NC_014121.1 | https://www.ncbi.nlm.nih.gov/nuccore/NC_014121.1 |
| Bacteroides fragilis NCTC 9343 [20] | NC_003228.3 | https://www.ncbi.nlm.nih.gov/nuccore/NC_003228.3 |
| Haemophilus influenzae F3031 [21] | NC_014920.1 | https://www.ncbi.nlm.nih.gov/nuccore/NC_014920.1 |
| Enterococcus faecium DO [22] | NC_017960.1 | https://www.ncbi.nlm.nih.gov/nuccore/NC_017960.1 |
| Enterobacter aerogenes KCTC 2190 [23] | NC_015663.1 | https://www.ncbi.nlm.nih.gov/nuccore/NC_015663.1 |
| Klebsiella oxytoca KCTC 1686 [24] | NC_016612.1 | https://www.ncbi.nlm.nih.gov/nuccore/NC_016612.1 |
| Proteus mirabilis BB2000 [25] | NC_022000.1 | https://www.ncbi.nlm.nih.gov/nuccore/NC_022000.1 |

## List of query organism and target organisms used for the padlock probe target search. Listed are also their respective accession numbers and web link to where the FASTA files can be retrieved.
