# Supplementary material for: microTaboo: a general and practical solution to the k-disjoint problem
Source: BMC Bioinformatics. 2017 May 2;18:228. doi: 10.1186/s12859-017-1644-6 (PMC5414201; doi:10.1186/s12859-017-1644-6)
Supplement: Supplementary file 2 — Section 1 - Results, Section 2 - Runtime Comparisons. (DOCX 27 kb) [file 12859_2017_1644_MOESM2_ESM.docx]

Additional File 1

# Section 1 - Results.

## 1.1 Padlock Probe target search

The experiment was performed with the following parameters: *W* = 30, *k* = 5, with the *k-disjoint* option (*d*). *Escherichia coli* O157 [1] was placed in a folder A, the “taboo” organisms in a folder B and the results were to be saved in a folder R.

FASTA files for all organisms were retrieved from the National Center for Biotechnology Information (NCBI) database. The names of the organisms as well as their accession numbers and web links are listed in Supplementary Table 1.

## 1.2 Point mutation detection

This run was performed with the following parameters: *W*=30, *k =* 0, with the *k-intersection* option (*i*). For each run, the FASTA file was point mutation had occurred were placed in folder A while the original FASTA file was placed in folder B.

FASTA files for the *Tobacco leaf curl Japan virus* [26] *(TbLCJV)* and *Escherichia coli 0157* *Sakai chr.* (same as above) were retrieved from the NCBI database with accession numbers and web links as shown in Supplementary Table 2.

The FASTA file for *Candida albicans* [27] was retrieved from the Candida genome database, assembly 22 at: <http://www.candidagenome.org/download/sequence/C_albicans_SC5314/Assembly22/current/>

However, as the sequence files are updated weekly, the particular FASTA file used in our experiment might not be the same as the current one. Therefore, please visit our github-page for the correct version at:

https://github.com/MohammedAlJaff/microTaboo

## 1.3 Inversion detection and virus incorporation

### 1.3.1 Inversion Detection

The inversions were carried out as follows:

**Lines inverted in FASTA file (without substitutions):**

*E. coli:* Line 331

*TbLCJV:* Line 14

*Saccharomyces cerevisiae:* Lines 10 (chr I), 3852 (chr II) and 48230 (chr V)

microTaboo’s parameters were: *W*=30, *k*=0 and *k-disjoint* option (*d*)

**Lines inverted with substitutions:**

*S. cerevisiae:* Lines 17 (chr I), 3849 (chr II), 48217 (chr V), 57835 (chr VI)

Inverted line 17 (no substitution)

CGAGCCACTTATTTCTCACAATGCTGTCACTACACTGTATAATAAGATTACATACCGGAC

Inverted line 3849 (1 substitution)

GCTCTAGTCGAACGCGACCCTCAATGGGGTAGATTTCACGGCGTAACCTGCCGTAAGGAC

|

GCTCTAGTCGTACGCGACCCTCAATGGGGTAGATTTCACGGCGTAACCTGCCGTAAGGAC

Inverted line 48217 (2 substitutions)

CAGGAATGCCGTCCAATGCGGCACTTTAGATGGGGTAACTCCCAGCGCATGCTGATCTCG

| |

CAGGAATGCGGTCCAATGCGGCACTATAGATGGGGTAACTCCCAGCGCATGCTGATCTCG

Inverted line 57835 (3 substitutions)

ACGATCTCTCTTTCTTTGACTTTCGTGTTAAAGGGTTATGAAGGCTCTTATACTTACAGA

| | |

AGGATCTCTCTTTCTTAGACTTTCGTGTTAAAGGGTAATGAAGGCTCTTATACTTACAGA

microTaboo’s parameters were: *W*=60, *k*=0 and *k-disjoint* option (*d*)

### 1.3.2 Virus incorporation

70 bp regions from *TbLCJV* where inserted into *S. cerevisiae* in the following way, with the original region and region with substitutions shown:

Inserted line 16 of *TbLCJV* (no substitutions) into line 12 of *S. cerevisiae*

AATGCTTTATTATTGTATATGGCTTGTACACATGCCAGTAATCCTGTGTATGCTACTCTAAAGATCAGAG

Inserted line 26 of *TbLCJV* (1 substitution) into line 3851 of *S. cerevisiae*

### GGTGTATGATGACGTTGGGCCTGGATTGCAGAGGAAGATTGTTGGGATACCACCTTTAATTTGAATTGGT

### |

### GGTGTATGAAGACGTTGGGCCTGGATTGCAGAGGAAGATTGTTGGGATACCACCTTTAATTTGAATTGGT

Inserted line 30 of *TbLCJV* (2 substitutions) into line 17406 of *S. cerevisiae*

TCAATCACAATGCTTATGGGCCTCCACGGCCGCGCAGCGGAATCCCTGGCGTTCTCTGCAGCCCATTCCT

| |

TCAATCACAATGCTTATGGGGCTCCACGGCCGCGCAGCGGAATCCCTGGCGATCTCTGCAGCCCATTCCT

Inserted line 6 of *TbLCJV* (3 substitutions) into line 22692 of *S. cerevisiae*

ATACTCGTTGTTCGTGCGAAGAACTATGTCGAAGCGTCCTGCAGATATAGGGATTTTCACTCCCGTCTCC

| | |

ATACTCGTTGTTCGAGCGAAGAACTATGTCGAAGCCTCCTGCAGATATAGGGATTTTCACTCCCGTCACC

microTaboo’s parameters were *W*=70, *k = 3* and *k-intersect* option (*i*)

FASTA files for the inversion detection run and virus incorporation run were retrieved from the NCBI database with accession numbers and web links as shown in Supplementary Table 3.

## 1.4 Candidate CRISPR-target mining

As this run was supposed to find self-relative unique sequences, the same FASTA files were placed in query folder A and “taboo” folder B. To reduce memory consumption, the chromosomal FASTA files of *Mus musculus* [29] were chopped into smaller files of 50MB each before being placed in the “taboo” folder B. The parameters used were as follows: *W = 20*, *k* was iterated over 0,1 and 2, the *k*-disjoint option (*d*) was used and the parameter *m* which is required when self-relative unique sequences are supposed to be found.

The FASTA file for *Candida albicans* was retrieved from the Candida genome database, assembly 22 at: <http://www.candidagenome.org/download/sequence/C_albicans_SC5314/Assembly22/current/>

However, as the sequence files are updated weekly, the particular FASTA file used in our experiment might not be the same as the current one. Therefore, please visit our github-page for the correct version at:

https://github.com/MohammedAlJaff/microTaboo

The genome FASTA files for *Drosophila Melanogaster* [30] were retrieved from Berkeley Drosophila Genome Projects, release 5, at:

<http://www.fruitfly.org/sequence/release5genomic.shtml>

The *M. musculus* genome was retrieved from the NCBI database at:

<https://www.ncbi.nlm.nih.gov/genome/52>

# Section 2 – Runtime Comparisons

## 2.1 Runtime comparison, microTaboo vs BLAST vs Suffix array

The setup for the BLAST analysis were based on two steps:

*i)* Creating a database of the taboo organism(s) using BLASTs tool for creating databases from FASTA files:

-makeblastdb -in tabooOrganism.fna -parse_seqids -dbtype nucl

*ii)* Divide the FASTA file of the query organism into words of length *W*. This would make the BLAST runs produce results similar to those obtained from microTaboo and make the two comparable. The parameters for each BLAST run were as follows:

-word_size 5, -max_hsps 1, -gapopen 100, -gapextend 100, perc_identity X.

These parameters were chosen in an attempt to emulate microTaboo as much as possible. All tests were conducted using blast+, 2.4.0. Comparisons were run for randomly generated sequences as well as for the two organisms, *Enterobacteria phage lambda* [31] and *E. coli* str. K12 [32]. Both genomes were retrieved from NCBI’s database at:

*Enterobacteria phage lambda* (J02459.1), <https://www.ncbi.nlm.nih.gov/nuccore/215104/>

*E. coli* str. K12. (NC_000913.3), https://www.ncbi.nlm.nih.gov/nuccore/556503834

For the suffix array, we used the implementation provided by Cola (<https://github.com/nedaz/cola)>, adjusting the parameter –S, which determines the minimum partial exact match length according to the pigeon hole principle.

Result coverage, i.e. the amount of sequences found by each run is presented in Supplementary Table 4 and was performed on a subset of the results sets obtained for the runtime comparison between *Enterobacteria phage lambda* and *E. coli* str. K12. Runtime results are shown in Supplementary Table 5.

**2.2 Runtime comparison, microTaboo vs exact string matching algorithms**

We compared the exact-match performance of microTaboo against three exact string matching algorithms: Boyer-Moore, Rabin-Karp, and Knuth-Morris-Pratt. The data set was the same as the *Enterobacteria phage lambda* vs *E.coli* for the BLAST and suffix array comparisons. The results for the runtimes are shown in Supplementary Table 6. Links to the java algorithm implementations used can be found below.

The source code for the exact string-matching algorithms were obtained from:

Boyer-Moore:

https://store.fmi.uni-sofia.bg/fmi/logic/vboutchkova/sources/BoyerMoore.java

Rabin-Karp:

http://www.sanfoundry.com/java-program-rabin-karp-algorithm/

Knuth-Morris-Pratt:

http://www.sanfoundry.com/java-program-knuth-morris-pratt-algorithm/

# References

1. Hayashi T. *et al.* Complete genome sequence of enterohemorrhagic Escherichia coli O157:H7 and genomic comparison with a laboratory strain K-12. 2001:DNA Res. 2001;8(1):11-22

2. Xiong Y. *et al*. A novel Escherichia coli O157:H7 clone causing a major hemolytic uremic syndrome outbreak in China. PLoS ONE. 2012;7(4):E36144

3. Chaudhuri R.R. *et al*. Complete genome sequence and comparative metabolic profiling of the enteroaggregative Escherichia coli strain 042. PLoS ONE, 2010;5(1):E8801

4. Nash J.H. *et al*. Genome sequence of adherent-invasive Escherichia coli and comparative genomic analysis with other E. coli pathotypes. BMC Genomics, 2010;11(1):667

5. Johnson S.L. *et al*. NCBI Reference Sequence: NC_018658.1. (Direct Submission), Submitted (2012-Feb-02) Genome Science B6, Los Alamos National Laboratory, Los Alamos, USA

6. Ogura Y. *et al*. Comparative genomics reveal the mechanism of the parallel evolution, PNAS. 2009;106(42):17939-17944

7. Iguchi A. *et al*. Complete genome sequence and comparative genome analysis of enteropathogenic Escherichia coli O127:H6 strain E2348/69. J. Bacteriol., 2009;191(1):347-354

8. Cooper K.K. *et al*. Complete Genome Sequences of Two Escherichia coli O145:H28 Outbreak. Genome Announc., 2014;2(3):e00482-14

9. Eppinger M. *et al*. Genomic anatomy of Escherichia coli O157:H7 outbreaks. PNAS., 2011;108(50):20142-20147

10. Winsor G.L. *et al*. Enhanced annotations and features for comparing thousands of Pseudomonas genomes in the Pseudomonas genome database. Nucleic Acids Res., 2016;doi:10.1093/nar/gkv1227.

11. Ferretti J.J. *et al*. Complete genome sequence of an M1 strain of Streptococcus pyogenes. PNAS. 2001;98(8):4658-4663

12. Hoskins J.A. *et al.* Genome of the bacterium Streptococcus pneumoniae strain R6. J. Bacteriol., 2001;183(19):5709-5717

13. Shankar, N. *et al*. Modulation of virulence within a pathogenicity island in vancomycin-resistant Enterococcus faecalis. *Nature*, 2002;417(6890):746-50

14. Nelson K.E. *et al.* Whole genome comparisons of serotype 4b and 1/2a strains of the food-borne pathogen Listeria monocytogenes reveal new insights into the core genome components of this species. Nucleic Acids Res., 2004;32(8):2386-2395

15. Wu K.M. *et al*. Genome sequencing and comparative analysis of Klebsiella pneumoniae NTUH-K2044, a strain causing liver abscess and meningitis. J. Bacteriol., 2009;191(14):4492-4501

16. McClelland M. *et al*. NCBI Reference sequence: NC_009792.1 (Direct Submission). Submitted (29-AUG-2007) Genetics, Genome Sequencing Center, St. Louis, MO. USA

17. Iacono M. *et al*. Whole-genome pyrosequencing of an epidemic multidrug-resistant Acinetobacter baumannii strain belonging to the European clone II group. Antimicrob. Agents Chemother., 2008;52(7):2616-2625

18. Lucas S. *et al*. NCBI Reference sequence: NC_012803.1 (Direct Submission).

Submitted (27-MAY-2009) US DOE Joint Genome Institute, CA, USA

19. Ren Y. *et al*. Complete genome sequence of Enterobacter cloacae subsp. cloacae

type strain ATCC 13047. J. Bacteriol., 2010:192(9):2463-2464

20. Cerdeno-Tarraga A.M. *et al*. Extensive DNA inversions in the B. fragilis genome control variable gene expression. Science, 2005;307(5714):1463-1465

21. Strouts F.R. *et al*. Lineage-specific virulence determinants of Haemophilus influenzae biogroup aegyptius. Emerging Infect. Dis., 2012;18(3):449-457

22. Qin X. *et al*. Complete genome sequence of Enterococcus faecium strain TX16 and comparative genomic analysis of Enterococcus faecium genomes. BMC Microbiol., 2012;12(1):135

23. Shin S.H. *et al*. Complete genome sequence of Enterobacter aerogenes KCTC 2190. J. Bacteriol., 2012;194(9):2373-2374

24. Shin S.H. *et al*. Complete genome sequence of Klebsiella oxytoca KCTC 1686, used in production of 2,3-butanediol. J. Bacteriol., 2012;194(9):2371-2372

25. Pearson M.M. *et al*. Complete genome sequence of uropathogenic Proteus mirabilis, a master of both adherence and motility. J. Bacteriol., 2008;190(11):4027-4037

26. Brown J.K. *et al*. Emergence of a New Cucurbit-Infecting Begomovirus Species Capable of Forming Viable Reassortants with Related Viruses in the Squash leaf curl virus Cluster. Phytopathology, 2002;92(7):734-742

27. Skrzypek, M. S. *et al.* Candida Genome Database (CGD). http://www.candidagenome.org/. Last accessed 6 April 2017.

28. Saccharomyces Genome Database (SGD). Stanford University, Stanford, CA.

http://www.yeastgenome.org. Last Accessed 6 April 2017.

29. Mouse Genome Sequencing Consortium *et al.* Initial sequencing and comparative analysis of the mouse genome. *Nature*, 2002;420(6915):520-62

30. Berkeley Drosophila Genome Project (BDGP). Berkeley University, Berkeley, CA, 2006. http://www.fruitfly.org/sequence/release5genomic.shtml. Last Accessed 6 April 2017.

31. Wu, R. and Taylor, E. Nucleotide sequence analysis of DNA. II. Complete nucleotide sequence of the cohesive ends of bacteriophage lambda DNA. *J. Mol. Biol.,* 1971;57(3):491-511

32. Blattner, F. R. *et al.* The complete genome sequence of Escherichia coli K-12. *Science*, 1997;277(5331):1453-62
