# Supplementary material for: microTaboo: a general and practical solution to the k-disjoint problem
Source: BMC Bioinformatics. 2017 May 2;18:228. doi: 10.1186/s12859-017-1644-6 (PMC5414201; doi:10.1186/s12859-017-1644-6)
Supplement: Supplementary file 3 — Point mutation detection. (DOCX 39 kb) [file 12859_2017_1644_MOESM3_ESM.docx]

Additional file 3: Table S2. Point mutation detection

| **Organism** | **Accession**  **number** | **Web link** |
| --- | --- | --- |
| Tobacco leaf curl Japan virus | NC_004645.1 | https://www.ncbi.nlm.nih.gov/nuccore/NC_004645.1 |
| Escherichia coli O157:H7 str. Sakai Chromosome | NC_002695.1 | https://www.ncbi.nlm.nih.gov/nuccore/NC_002695.1 |

List of organisms used in the point mutation detection run, including accession numbers and web links.
