# Supplementary material for: microTaboo: a general and practical solution to the k-disjoint problem
Source: BMC Bioinformatics. 2017 May 2;18:228. doi: 10.1186/s12859-017-1644-6 (PMC5414201; doi:10.1186/s12859-017-1644-6)
Supplement: Supplementary file 4 — Inversion detection and virus incorporation. (DOCX 54 kb) [file 12859_2017_1644_MOESM4_ESM.docx]

Additional file 4: Table S3. Inversion detection and virus incorporation

| **Organism** | **Accession**  **number** | **Web link** |
| --- | --- | --- |
| Tobacco leaf curl Japan virus | NC_004645.1 | <https://www.ncbi.nlm.nih.gov/nuccore/NC_004645.1> |
| Escherichia coli O157:H7 str. Sakai Chromosome | NC_002695.1 | <https://www.ncbi.nlm.nih.gov/nuccore/NC_002695.1> |
| Saccharomyces cervisiae S288c [28] | - | <https://www.ncbi.nlm.nih.gov/genome/15> |

Organisms used in the inversion detection run and virus incorporation run, including accession number and web link. Accession number is not included for *Saccharomyces cervisiae* as the accession numbers are for individual chromosomes rather than the entire genome.
