# Supplementary material for: microTaboo: a general and practical solution to the k-disjoint problem
Source: BMC Bioinformatics. 2017 May 2;18:228. doi: 10.1186/s12859-017-1644-6 (PMC5414201; doi:10.1186/s12859-017-1644-6)
Supplement: Supplementary file 5 — Runtime comparisons -microTaboo vs. BLAST and a suffix array method. (DOCX 39 kb) [file 12859_2017_1644_MOESM5_ESM.docx]

**Additional file 5: Table S4.** Result coverage comparison – BLAST vs microTaboo

| **W** | **Size of BLAST vs microTaboo** |
| --- | --- |
| 20 | 20% |
| 40 | 93% |
| 60 | 94% |
| 100 | 95% |

Result coverage is calculated as the fraction of sequences found between both software’s. While microTaboo obtained many more results for a short sequence length (20), the results were much more similar as word length increased. Mismatch threshold was set to 3 in all cases.
