# Supplementary material for: microTaboo: a general and practical solution to the k-disjoint problem
Source: BMC Bioinformatics. 2017 May 2;18:228. doi: 10.1186/s12859-017-1644-6 (PMC5414201; doi:10.1186/s12859-017-1644-6)
Supplement: Supplementary file 6 — Result coverage comparison — BLAST vs microTaboo. (DOCX 86 kb) [file 12859_2017_1644_MOESM6_ESM.docx]

Additional file 6: Table S5. Runtime comparisons –microTaboo vs. BLAST and a suffix array method

|  | **Random** | **sequences** | ***Lamda phage vs E.coli*** | | |
| --- | --- | --- | --- | --- | --- |
| **W/k** | **BLAST** | **microTaboo** | **BLAST** | **Suffix array** | **microTaboo** |
| **W = 20** |  |  |  |  |  |
| **k = 0** | 196 | 26 | 329 | 283 | 29 |
| **k = 3** | 196 | 39 | 323 | -^*^ | 42 |
| **k = 5** | 206 | - | 328 | -^*^ | 36 |
| **W = 40** |  |  |  |  |  |
| **k = 0** | 458 | 35 | 889 | 228 | 33 |
| **k = 3** | 459 | 56 | 875 | 4121 | 58 |
| **k = 5** | 462 | 321 | 872 | -^*^ | 316 |
| **W = 60** |  |  |  |  |  |
| **k = 0** | 779 | 36 | 1555 | 186 | 44 |
| **k = 3** | 777 | 57 | 1550 | 691 | 73 |
| **k = 5** | 818 | 399 | 1581 | 4121 | 422 |
| **W = 80** |  |  |  |  |  |
| **k = 0** | 1099 | 40 | 2608 | 164 | 49 |
| **k = 3** | 1108 | 64 | 2764 | 283 | 84 |
| **k = 5** | 1166 | 445 | 2533 | 2386 | 501 |
| **W = 100** |  |  |  |  |  |
| **k = 0** | 1506 | 43 | 3409 | 123 | 55 |
| **k = 3** | 1562 | 73 | 3425 | 258 | 96 |
| **k = 5** | 1531 | 559 | 3708 | 395 | 621 |

Second and third columns show the runtime in seconds for BLAST and microTaboo on a set of randomly generated sequences. Fourth, fifth and sixth columns show the runtime of BLAST, the suffix array implemented in the Cola package, and microTaboo, in seconds for a dataset consisting of *Enterobacteria phage lambda* and *E. coli str K12.* The asterisk (“*”) indicates cases in which program did not finish within two hours of runtime. The leftmost column indicates what parameters were used, with sequence length denoted with W and mismatch threshold by k**.**
