# Supplementary material for: microTaboo: a general and practical solution to the k-disjoint problem
Source: BMC Bioinformatics. 2017 May 2;18:228. doi: 10.1186/s12859-017-1644-6 (PMC5414201; doi:10.1186/s12859-017-1644-6)
Supplement: Supplementary file 7 — Runtime comparisons for exact string matching algorithms. (DOCX 50 kb) [file 12859_2017_1644_MOESM7_ESM.docx]

**Additional file 7: Table S6.** Runtime comparisons for exact string matching algorithms

| **W** | **microTaboo** | **Boyer-Moore** | **Rabin-Karp** | **Knuth-Morris-Pratt** |
| --- | --- | --- | --- | --- |
| 20 | 29 | 140 | 794 | 387 |
| 40 | 33 | 122 | 1200 | 391 |
| 60 | 44 | 114 | 1453 | 379 |
| 80 | 49 | 108 | 1624 | 380 |
| 100 | 55 | 105 | 1775 | 382 |

First column shows the word lengths (W) used for each run. Second to fifth column shows the runtime in seconds for each algorithm. The comparisons were performed for exact matching (i.e. *k* = 0 for microTaboo). The dataset was the same as above (section 2.1), i.e. *Enterobacteria phage lambda* and *E. coli* *str K12.* In each case, microTaboo was the fastest, but with increasing runtime depending on *W*. The Rabin Karp method exhibited similar characteristics, while Knuth-Morris-Pratt is constant, and Boyer-Moore decreased runtime with increasing *W*.
